# Molecules and the Eigenstate Thermalization Hypothesis

**DOI:** 10.3390/e20090673

**Published:** 2018-09-05

**Authors:** David M. Leitner

**Affiliations:** Department of Chemistry, University of Nevada, Reno, NV 89557, USA; dml@unr.edu; Tel.: +1-775-784-1968

**Keywords:** vibrational state space, local random matrix theory, many-body localization

## Abstract

We review a theory that predicts the onset of thermalization in a quantum mechanical coupled non-linear oscillator system, which models the vibrational degrees of freedom of a molecule. A system of *N* non-linear oscillators perturbed by cubic anharmonic interactions exhibits a many-body localization (MBL) transition in the vibrational state space (VSS) of the molecule. This transition can occur at rather high energy in a sizable molecule because the density of states coupled by cubic anharmonic terms scales as *N*^3^, in marked contrast to the total density of states, which scales as exp(*aN*), where *a* is a constant. The emergence of a MBL transition in the VSS is seen by analysis of a random matrix ensemble that captures the locality of coupling in the VSS, referred to as local random matrix theory (LRMT). Upon introducing higher order anharmonicity, the location of the MBL transition of even a sizable molecule, such as an organic molecule with tens of atoms, still lies at an energy that may exceed the energy to surmount a barrier to reaction, such as a barrier to conformational change. Illustrative calculations are provided, and some recent work on the influence of thermalization on thermal conduction in molecular junctions is also discussed.

## 1. Introduction

**Figure 1:**
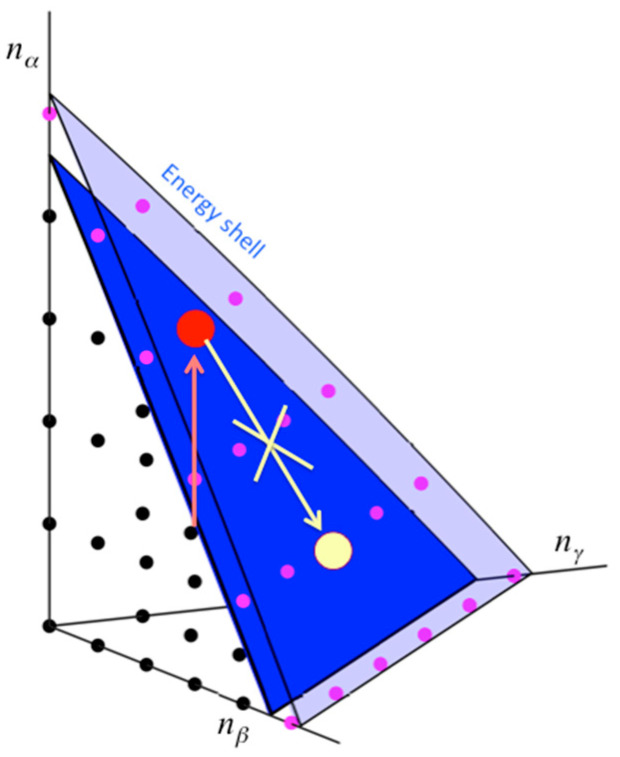
Vibrational states of a molecule, modeled as a quantum mechanical system of many coupled non-linear oscillators. Each state depends on the number of vibrational quanta, *n*, that occupy the modes of the molecule, three of which are plotted. If mode *α* is excited (red arrow) so that the molecule is brought to a new state (red dot), then thermalization, if it occurs, will lead to the equilibrium distribution for **n** (yellow dot). However, if the anharmonic coupling or the local density of resonantly coupled states of the molecule is sufficiently small, relaxation occurs only very slowly, or not at all if the molecule is isolated, an example of many-body localization (MBL), which is indicated in the figure as the absence of a pathway from the initially excited state to the thermalized state. In that case the ETH is invalid. Precise criteria for validity of the ETH for molecules are provided in the text. Reprinted with permission from Pandey, H.D. and Leitner, D.M. “Influence of thermalization on thermal conduction through molecular junctions: Computational study of PEG oligomers,” *J. Chem. Phys.* **147**, 084701, Copyright (2017), American Institute of Physics.

The Eigenstate Thermalization Hypothesis (ETH) provides justification for replacing time averages with ensemble averages of closed quantum mechanical systems [1,2,3,4]. For chemists, the ETH offers at least partial rationale for the application of microcanonical transition state theory (TST) to predict the rate of, say, the conformational change of a molecule. However, concerns about whether molecules thermalize under their own dynamics have long been raised [5,6,7,8,9]. In the following, we think of a molecule as a quantum mechanical system of coupled nonlinear oscillators. The system of uncoupled oscillators is integrable and does not obey the ETH. If, upon perturbation, the ETH is valid for the coupled oscillator system, i.e., molecule, the time-average for a molecule exploring the vibrational state space (VSS, sketched in Figure 1) can be replaced by an ensemble average. The molecule, prepared out of equilibrium, relaxes to equilibrium and stays there, with few significant deviations, as time progresses. However, the ETH is often not satisfied for molecules [9]. The vibrational eigenstates of even a large molecule are often localized in the VSS, an example of many-body localization (MBL) [10]. In this article, we review criteria for MBL in the VSS of a large molecule, and discuss implications for the validity of the ETH for molecules, its application in reaction rate theory and thermal transport through molecules.

Consider a molecule that has been excited to a particular state of the VSS, which could occur through collision with another molecule or photoexcitation with a laser. This is illustrated in Figure 1, where the VSS is depicted in terms of three of the oscillators of the molecule. The full Hamiltonian and definition of the VSS will be provided in Section 2. For now, we can think of each zero-order state of the VSS as represented by a lattice point, each corresponding to the set of the number of quanta occupying the uncoupled oscillators. We excite the molecule to a state out of equilibrium, illustrated by one of the lattice points in Figure 1. Since the states are coupled by anharmonic interactions, the system is no longer integrable and may thermalize. However, coupling in the VSS is local. If we, e.g., consider for now only cubic anharmonic interactions, sites that lie at most a distance 3 positions away from the state in which the molecule was prepared are directly coupled. Even if the total density of states of the molecule is quite large, relatively few states are coupled by the cubic anharmonic interactions. More specifically, though the total density of states increases as exp(*aN*), where *N* is the number of oscillators and *a* is a constant, the local density of states coupled by the cubic anharmonic interactions increases only as *N*^3^. The magnitude of the coupling needed for the ETH to be valid is therefore much larger than if all zero-order states of the VSS were coupled. We cannot, of course, simply truncate the interactions at third-order in the anharmonicity, since higher-order terms, while relatively small, are numerous and become more important in establishing quantum ergodicity as *N* increases. Nevertheless, the locality of coupling remains important.

The failure of many isolated molecules to thermalize under their own dynamics has been observed experimentally for a long time. A consequence of this is that rates of chemical reactions, such as conformational change of organic molecules with of order 10 to 100 degrees of freedom, are not well predicted by microcanonical TST [9,11,12,13,14,15,16,17,18,19,20,21]. In this approach, molecules may be prepared in any number of states via collision or photoexcitation. The rate of reaction of a molecule that is taken to be isolated between collisions is estimated by identifying states from which reaction occurs, and calculating the probability that the molecule occupies such states assuming an equilibrium ensemble. The product of that probability and the rate to form product given that the molecule occupies such a state is the microcanonical TST reaction rate. The formalism for this calculation, named for four of its developers and called RRKM theory (Rice–Ramsperger–Kassel–Marcus theory) [22,23,24], continues to enjoy wide use [25]. We shall point out below one of a number of important cases where the theory fails to predict the reaction rate to illustrate how the breakdown of the ETH and the emergence of MBL in molecules manifest themselves in chemistry.

With that practical consideration in mind, we seek criteria for which the ETH is valid for molecules. In the following section, we review the theory of energy flow in a quantum mechanical system of many coupled oscillators and MBL in the Fock space of a molecule. The theory of localization in the VSS, introduced by Logan and Wolynes [7], was later generalized to address higher-order and off-resonant coupling [26,27,28,29,30], and has recently been the subject of a review [9]. Related theoretical work on localization in the Fock space of a many-electron system developed later yields a similar picture [31]. In Section 2, we begin by modeling a molecule as a nonlinear oscillator system coupled by cubic anharmonic interactions. We truncate the anharmonicity at cubic terms to illustrate the role of local interactions in the VSS, criteria for localization and violation of the ETH in molecules. We then generalize to higher-order anharmonicity and examine the effect of higher order terms on localization in the VSS. In Section 3, we illustrate the importance of thermalization in molecules in chemical reaction kinetics, where we consider the example of the kinetics of conformational change of an organic molecule with effectively 60 vibrational modes. In Section 4, we discuss recent work on thermal transport through molecular junctions, where the issue of thermalization also arises. Conclusions are presented in Section 5.

## 2. Criteria for Quantum Ergodicity in Molecules

The coupled nonlinear oscillator Hamiltonian is *H* = *H*_0_ + *V*,

(1a) $$H_{0}=\sum_{\alpha =1}^{N}\varepsilon_{\alpha }({\hat{n}}_{\alpha })$$

(1b) $$V=\frac{1}{3!}\sum_{\alpha ,\beta ,\gamma }\phi_{\alpha \beta \gamma }\left(b_{\alpha }^{+}+b_{\alpha }\right)\left(b_{\beta }^{+}+b_{\beta }\right)\left(b_{\gamma }^{+}+b_{\gamma }\right)$$

where the sum in Equation (1b) excludes *α* = *β* = *γ*. The zero-order Hamiltonian *H*_0_ consists of a sum over the energies of the *N* nonlinear oscillators, where each oscillator has frequency $\omega_{\alpha }(n_{\alpha })=\hbar^{-1}\partial \varepsilon_{\alpha }/\partial n_{\alpha }$, and nonlinearity $\omega_{\alpha }^{\prime }(n_{\alpha })=\hbar^{-1}\partial \omega_{\alpha }/\partial n_{\alpha }$, and the number operator is defined by ${\hat{n}}_{\alpha }=b_{\alpha }^{+}b_{\alpha }$. The potential in Equation (1) includes cubic anharmonic coupling. We will modify the Hamiltonian to address anharmonicity of higher order later.

We take the set of zero order energies, $\left\{\varepsilon_{\alpha }\right\}$, and the cubic anharmonic coefficients, $\left\{\phi_{\alpha \beta \gamma }\right\}$, to be random variables with suitable distributions. For example, the distribution of zero-order energies will be determined in part by the distribution of normal mode frequencies of the molecule. Equation (1) represents the perturbation of the integrable system, *H*_0_, and we seek criteria for which the perturbation leads to an ergodic system, where the eigenstates of *H* satisfy the ETH. Alternatively, we may find the eigenstates of this many-body system to be localized. Because of the locality of the coupling in the vibrational state space we refer to the theory as Local Random Matrix Theory (LRMT). The local coupling in this random matrix model is far more restrictive than, say, a banded random matrix (BRM) ensemble [32,33,34], which has been used to examine criteria for quantum ergodicity [1].

LRMT has the form of a tight-binding picture in the VSS with random site energies. Each site in the VSS is an eigenstate |j〉 of the uncoupled Hamiltonian, *H*_0_. If there are $n_{\alpha }^{\left(j\right)}$ quanta in oscillator *α* in state |j〉, then this zero-order state is given by $|j\rangle =|n_{1}^{\left(j\right)},\;n_{2}^{\left(j\right)},\;\cdots ,\;n_{N}^{\left(j\right)}\rangle$ for the *N* oscillator system. In this basis, given the locality of the coupling between the zero-order states, the coupled many-oscillator Hamiltonian is equivalent to a tight-binding Hamiltonian,

(2) $$H=\sum_{j}\varepsilon_{j}|j\rangle \langle j|+\sum_{j,k(k\neq j)}V_{j,k}|j\rangle \langle k|$$

where the sums run over all sites in the VSS. The problem of quantum energy flow and ergodicity in the VSS of a molecule thereby resembles the problem of single-particle quantum transport on a many-dimensional disordered lattice. Theoretical approaches to address the condensed phase problem can be brought to bear on describing vibrational energy flow in molecules. Exploiting this connection, Logan and Wolynes identified criteria for localization in the VSS that occurs at a critical value of the product of the vibrational coupling and local density of states [7]. This is an example of many-body localization (MBL), which has been the focus of much attention in recent years [10,31,35,36,37,38,39,40].

The eigenstates $|E_{\lambda }\rangle$ of *H* are expressed in terms of |j〉 as

(3) $$|E_{\lambda }\rangle =\sum_{j}c_{j\lambda }|j\rangle$$

It is the nature of the coefficients, $\left\{c_{j\lambda }\right\}$, that is of interest. If the system *H* is brought to a state |j〉 by excitation with a laser, then whether or not the time average will resemble the microcanonical average depends on the coefficients, $\left\{c_{j\lambda }\right\}$. When an eigenstate of *H* is localized to state |j〉, the molecule will not relax to a Bose–Einstein distribution. More generally, consider an observable, *O*. Individual $\langle E_{\lambda }|\hat{O}|E_{\lambda }\rangle$ will not generally resemble the microcanonical average at energies close to the total energy of the molecule when an eigenstate of *H* is localized to state |j〉. When the ETH is valid, $\langle E_{\lambda }|\hat{O}|E_{\lambda }\rangle$ exhibits fluctuations that are exponentially small in *N*, so that any $\langle E_{\lambda }|\hat{O}|E_{\lambda }\rangle$ lies very close to the microcanonical average [1,2,3,4]. We expect the ETH to be valid if $|E_{\lambda }\rangle$ is extended and overlaps many zero-order states, where ${\left|c_{j\lambda }\right|}^{2}$ is of the order of the inverse of the number of states on the energy shell, which grows exponentially with *N*. This is the result one obtains when eigenvectors of *H* are those of random matrix ensembles such as the Gaussian Orthogonal Ensemble (GOE), which have been suggested to model spectral statistics of quantum systems for which the corresponding classical system exhibits chaos [41]. We seek to learn under what conditions we can make this association for molecules. 

The local density of states, $\rho_{3}(E)$, coupled to a zero-order state of the VSS via third-order anharmonic interactions scales with the number of oscillators, *N*, as *N*^3^. Let $\langle \left|V_{3}\right|\rangle$ be the average magnitude of an off-diagonal matrix element that couples two states via third-order anharmonic interactions. Detailed analysis reveals that the eigenstates of *H* are localized when [7],

(4) $$T(E)\equiv \frac{2\pi }{3}{\left(\langle \left|V_{3}\right|\rangle \;\rho_{3}\right)}^{2}<1$$

Indeed, one finds that the probability distribution of ${\left|c_{j\lambda }\right|}^{2}$ corresponds to the Porter–Thomas distribution when *T* > 1 [27], the result for the GOE. More specifically, we obtain for the probability distribution for $y\equiv {\left|c_{j\lambda }\right|}^{2}$, where the transition criterion has been satisfied [27],

(5) $$P_{y}(y)={\left(2\pi y\langle y\rangle \right)}^{-1/2}\exp\left(-\pi y/(2\langle y\rangle )\right)$$

The Porter–Thomas distribution is predicted to describe the distribution of ${\left|c_{j\lambda }\right|}^{2}$ for extended eigenstates of a many-body system when *T* > 1 as well as the eigenstates of a low-dimensional system at energies where the corresponding classical Hamiltonian exhibits chaotic dynamics [41,42]. We thus expect that when *T* > 1, the ETH should hold for molecules. The transition bears some resemblance to the resonance overlap criterion for classical chaos [41,43], and there indeed appears to be correspondence between the onset of chaos and quantum ergodicity found in computational studies of small systems [40,44,45]. We take, however, the coupled nonlinear oscillator system defined by Equation (1) to be relatively large, and the MBL transition may occur with on average less than one quantum per oscillator, away from the semiclassical limit.

In general, we need to consider higher-order anharmonic coupling. We thus generalize the Hamiltonian to include anharmonic coupling of arbitrary order. We express a coupled nonlinear oscillator Hamiltonian, *H* = *H*_0_ + *V*, as [9,26]

(6a) $$H_{0}=\sum_{\alpha =1}^{N}\varepsilon_{\alpha }({\hat{n}}_{\alpha })$$

(6b) $$V=\sum_{m}\;\prod_{\alpha }\Phi_{m}(b_{\alpha }^{+}{)}^{m_{\alpha }^{+}}{\left(b_{\alpha }\right)}^{m_{\alpha }}$$

where $m=\{m_{1}^{\pm },\;m_{2}^{\pm },\;\ldots \}$. As expressed by Equation (6b), *V* includes anharmonicity of all orders. For example, cubic anharmonicity includes terms where $\sum_{\alpha }m_{\alpha }^{\pm }$ is at most 3. For higher order anharmonicity, we assume [9,26]

(7) $$\Phi_{m}=\Phi_{3}\sigma^{p-3},\;p\geq 3$$

where $p=\sum_{\alpha }\left(m_{\alpha }^{+}+m_{\alpha }^{-}\right)$ and the sum is over all modes, which has been validated numerically for molecules [46]. As in a random matrix theory, the set of zero order energies, $\left\{\varepsilon_{\alpha }\right\}$, and coefficients, $\left\{\Phi_{m}\right\}$, are treated as random variables with suitable average and variance for each **m**.

To lowest order in the perturbation expansion [9], in which only direct resonant coupling is taken into account, one finds the criterion for localization [9,26],

(8) $$T(E)\equiv \frac{2\pi }{3}{\left[\sum_{Q=3}\langle |V_{Q}|\rangle \rho_{Q}\right]}^{2}<1$$

where *Q* represents an order in the anharmonic coupling. (Corrections due to higher-order terms in the perturbation expansion have been addressed and *T*(*E*) retains a form like Equation (8) [9,26,47].) In the limit where *N*/*σ* is of order 1 or less, terms beyond *Q* = 3 make little contribution to *T* and a potential truncated at cubic anharmonic interactions is sufficient. However, when *N* is very large, higher order terms need to be taken into account. More specifically, let *M* be the average occupation number of each oscillator and $\omega_{rms}$ the root-mean-square oscillator frequency; one finds that a good approximation to *T* is [26]

(9a) $$T(E)=\frac{2\pi }{3}\tilde{\phi^{2}}\sigma^{6}F^{2}(\kappa )$$

(9b) $$\kappa =\frac{2NM^{1/2}}{\sigma }$$

(9c) $$\tilde{\phi }=\Phi_{3}{\left(\pi \omega_{rms}\right)}^{-1}$$

(9d) $$F(\kappa )={\left(2\pi \right)}^{-1/2}\left(\sum_{Q=3}^{\infty }Q^{-1}{\left(\frac{\kappa e}{Q}\right)}^{Q}\right)$$

The order of anharmonicity that contributes most to the localization transition depends on values of *σ* and *N*. The term, *Q_m_*, that contributes most to the transition is *Q_m_* ≈ *κ* − 1, if this is greater than 3, and is otherwise the cubic anharmonic term [26]. Since often *σ* ≈ 10 [46], the model given by Equation (1), where the anharmonicity is truncated at third order, provides a reasonable predictor for the localization transition for many molecules, but for molecules with ≈ 10 or more atoms we need to consider higher order anharmonic terms to locate the localization transition. 

**Figure 2:**
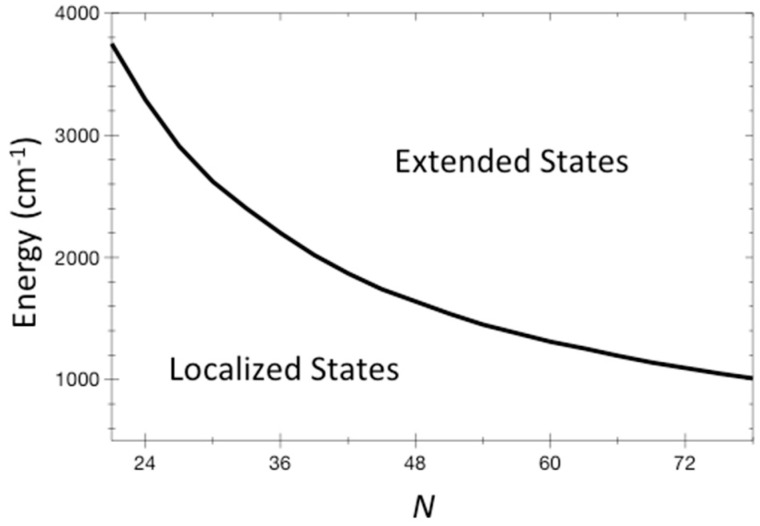
The energy where *T* = 1 for systems of *N* coupled non-linear oscillators is plotted for *N* from 21 to 78. This curve separates localized states at lower energy from extended states at higher energy. The ETH would be expected to be valid in the extended domain. Representative values for organic molecules are used for the distribution of vibrational frequencies and anharmonic couplings, and are given in the text.

We can plot the separation of localized from extended states in terms of parameters that represent vibrational properties of the molecule, and its energy. We do this in Figure 2, where we plot values of energy where *T* = 1 using Equation (9) at different *N* from 21 to 78. We have used as representative values $\Phi_{3}$ = 6.4 cm^−1^, $\omega_{rms}$ = 1127 cm^−1^, and *σ* = 10. (1 cm^−1^ corresponds to about 1.5 K). Some of these values have been obtained for the molecule stilbene, which is discussed in the next section, but they are anyway reasonable and representative of a sizable organic molecule. Importantly, the energies at which localized states are found can be higher than barriers to chemical reactions, even for large molecules. Reactions such as the change of structure of a molecule often occur over energy barriers on the order of 1000 cm^−1^. 

## 3. Quantum Ergodicity and the Kinetics of Conformational Change

It is convenient to assume thermalization in molecules when estimating rates of a chemical reaction, such as A→B, where *A* and *B* are two different structures of a molecule. In that case it may be possible to calculate the reaction rate using microcanonical transition state theory (TST). We assume that the reaction takes place at a fixed energy, *E*, that the molecule in structure *A* has enough energy for reaction to structure *B*, i.e., there is sufficient energy to surmount an energy barrier (or tunnel through it in a reasonable amount of time). If *A* and *B* are stable structures, the transition from one structure to another is a rare event that involves passage through a transition state, A*.

The reaction rate may be conveniently calculated using equilibrium statistical mechanics to determine the probability, P*, that a molecule in structure *A* is in a transition state, A*. The reaction rate is then calculated as the product of P* and the rate to pass from the transition state to structure *B*, which we will call $\nu_{A}$. The reaction rate, *k*(*E*), at energy *E* is thus $\nu_{A}P^{*}$. In practice, P* is very small and $\nu_{A}$ may be on the order of ps^−1^ or faster. There are well-developed methods for carrying out such calculations, notably RRKM theory, mentioned in Section 1, which have been applied to the calculation of rates of unimolecular reactions, those in which a molecule, after collision or photoexcitation, forms a new chemical species. We do not review RRKM theory here; the calculation is straightforward and has been applied for many years [48]. We refer to the RRKM theory microcanonical unimolecular reaction rate as $k_{RRKM}(E).$

In microcanonical TST there is an implicit assumption about relaxation times, which can be seen as follows: Consider the kinetic model $A\overset{{k^{\prime }}_{IVR}}{\underset{k_{IVR}}{↔}}A*\overset{\nu_{A}}{\to }B$. Here $k_{IVR}$ is the rate constant for what is often referred to as intramolecular vibrational redistribution (IVR), specifically for population transfer from a state of the transition region to a state outside the transition region. ${k^{\prime }}_{IVR}$ is the rate constant for population transfer from a state outside the transition region to a state in the transition region. Detailed balance gives ${k^{\prime }}_{IVR}=k_{IVR}P^{*}(E)$, where $P^{*}(E)$ is the probability that the reactant is in a transition state at energy, *E*. As already noted, the RRKM theory rate constant is $k_{RRKM}(E)=\nu_{A}(E)P^{*}(E)$. Applying the steady state approximation, the rate of isomerization from structure *A* to structure *B* is

(10) $$k(E)=\frac{k_{IVR}(E)}{k_{IVR}(E)+\nu_{A}(E)}k_{RRKM}(E)$$

This correction to $k_{RRKM}(E)$ includes the relaxation time, $1/k_{IVR}$, to equilibrate following depletion of a transition state due to reaction in the time $1/\nu_{A}$. Therefore, for the formalism of RRKM theory to provide an accurate estimate to the rate of conformational change both the ETH must be satisfied and the relaxation rate must be faster than the vibrational time for passing from a transition state to product. If the energy barrier to a reaction such as conformational change, or isomerization, lies below the energy at which the ETH is valid then RRKM theory clearly breaks down, and alternatives that address dynamics at these energies are required [49]. Even if the ETH is valid for that molecule at energies above the reaction barrier, the rate of reaction is often lower than predicted by RRKM theory due to the correction introduced in Equation (10). In that case transition rates, $k_{IVR}$, in the VSS need to be calculated separately to calculate the rate of reaction.

**Figure 3:**
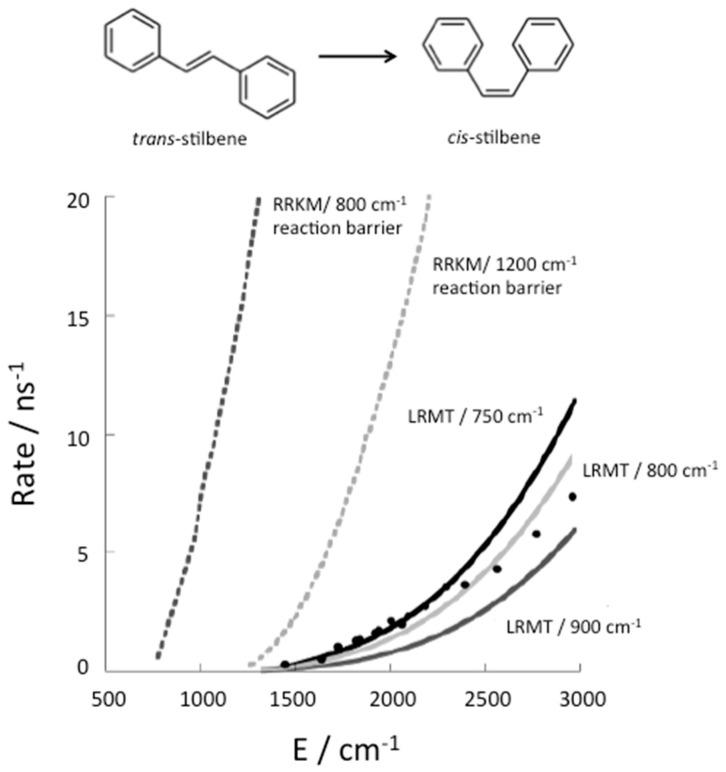
The RRKM theory (dotted curves) prediction for the reaction rate, in one case using an energy barrier to reaction of about 800 cm^−1^, as obtained by ab initio electronic structure calculations [15], and in another case using a 1200 cm^−1^ reaction barrier, as was assumed for some RRKM theory calculations [52]. Also plotted are corrections to the RRKM theory estimate to the rate using LRMT to establish where the quantum ergodicity threshold lies, as well as corrections due to the finite relaxation rate (solid line) for *trans*→*cis* isomerization of stilbene, shown at the top. Experimental results from Reference [13] are plotted as circles. LRMT rates are plotted for several barrier heights consistent with ab initio electronic structure calculations −750 cm^−1^ (black solid line), 800 cm^−1^ (light grey solid line), and 900 cm^−1^ (dark grey solid line). Reprinted with permission from Leitner, D.M., Levine, B., Quenneville, J., Martinez, T.J. and Wolynes, P.G. “Quantum energy flow and trans-stilbene photoisomerization: an example of a non-RRKM reaction,” *J. Phys. Chem. A* **107**, 10706–16. Copyright (2003) American Chemical Society.

To illustrate the influence of thermalization on chemical reaction kinetics, we consider the example of isomerization of stilbene, which has been very well studied experimentally [50]. Stilbene has 72 vibrational modes, but 12 of them are too high in frequency to contribute significantly to energy flow in the VSS, so that this molecule can be described effectively as a coupled 60-nonlinear oscillator system. Change in structure from *trans* to *cis*, shown in Figure 3, occurs efficiently with the absorption of a UV photon, which brings the molecule into an excited electronic state. In that state there is an energy barrier of about 800 cm^−1^ to conformational change from the *trans* structure, which has been determined by ab initio electronic structure calculations [15]. The rate of isomerization of *trans* stilbene has been measured in molecular beam experiments [51], in which the molecules collide only infrequently and can be prepared in states of well defined energy. If isomerization occurs more rapidly than fluorescence the rate can be detected.

Isomerization of *trans* stilbene is only observed at energies corresponding to about 1200 cm^−1^ or more vibrational energy in the excited electronic state [51]. Therefore 1200 cm^−1^ was assumed to be close to the barrier to reaction [52]. However, as seen in Figure 3, the RRKM theory rate calculated with that barrier was nevertheless found to be an order of magnitude greater than the rates that were measured [13] for the isomerization of *trans* stilbene in molecular beams. After ab initio electronic structure calculations set the reaction barrier at about 800 cm^−1^, it was recognized that the reason reaction was not observed at energies between about 800 and 1200 cm^−1^ is that the molecule is prepared experimentally in states that do not have access to transitions states, from where it can react, at least before it fluoresces back to the ground electronic state. Using LRMT, the quantum ergodicity threshold for stilbene was found to correspond to about 1200 cm^−1^ [15,16]. Therefore, above 1200 cm^−1^ the molecule has access to transition states regardless of the state in which it is prepared.

Above 1200 cm^−1^ the rate of conformational change from *trans* to *cis* is nevertheless lower than the rate predicted by RRKM theory, as seen in Figure 3, because the relaxation time in Equation (10) is rather slow compared to the time to form product from a transition state. When the relaxation rates are separately calculated [15,16,53], agreement between theory and experiment is very good, as seen in Figure 3, where three barriers to reaction close to 800 cm^−1^ are used in the calculations of the isomerization rate.

## 4. Thermalization and Thermal Transport in Molecules

Recently there has been much interest in the role of thermalization in thermal conduction through molecular junctions [54,55,56,57,58,59,60,61,62,63,64,65,66,67,68,69,70,71,72,73,74,75,76]. The study of thermal conduction through molecules of order 10 to 1000 atoms [14,77,78,79,80,81,82,83,84,85,86,87,88,89,90,91] has been motivated by the more general desire to control thermal transport at the nanoscale towards the design of nanoscale devices [92,93], small composite materials in which interfaces often mediate heat flow. Work on this problem has been driven by potential applications that include avoiding high concentrations of heat in small devices [92,93], thermoelectric applications [94], the possibility of thermal rectification at the nanoscale [61,95,96,97,98,99,100,101], and the contribution of thermal gradients to electron transfer [62,69,70].

**Figure 4:**
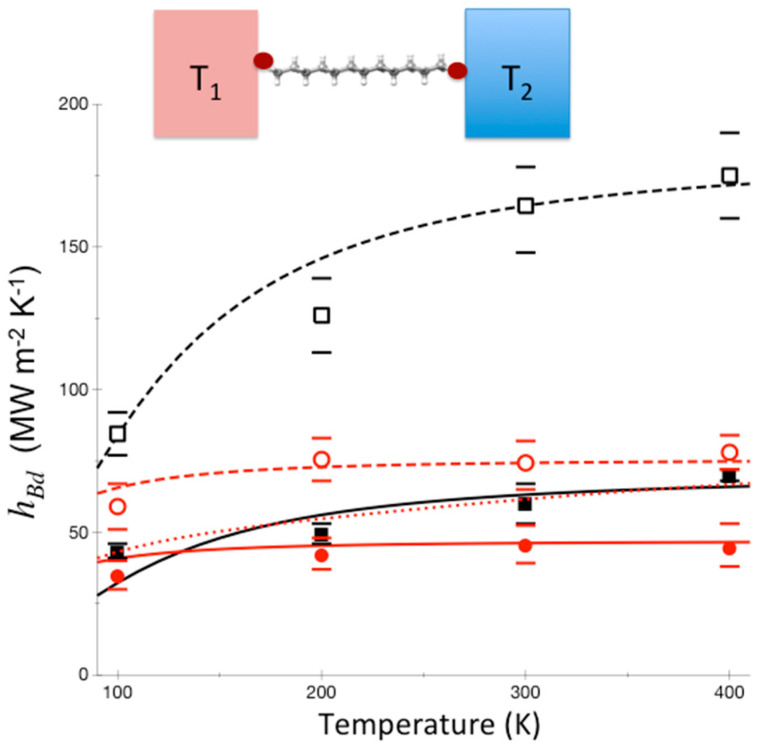
Calculations of thermal conductance between Au and sapphire (red) and between Al and sapphire (black) with carbon-chain molecular interface that is fluorinated (solid) or not (dashed), for temperatures from 100–400 K. The Landauer model is used, where thermalization is neglected, with a junction length of 2 nm and elastic scattering length calculated to be 0.55 nm [68]. For Au-perfluoroalkane-sapphire we compare with a calculation where thermalization is assumed to be rapid (red dotted curve), for comparison. Experimental results from Reference [60] are plotted (Au-sapphire red circles, Al-sapphire black squares, closed and open, respectively, for carbon-chain molecular interfaces that are fluorinated and not fluorinated) with reported error bars. The results of the experiments match those calculated using a Landauer picture, and are thus consistent with incomplete thermalization in these molecules to at least 2 nm. Reprinted with permission from Pandey, H.D. and Leitner, D. M., “Thermalization and Thermal Transport in Molecules,” *J. Phys. Chem. Lett.* **7**, 5062–5067. Copyright (2016) American Chemical Society.

On molecular scales of 1 nm, the energy diffusion picture and Fourier’s heat law break down, as revealed by numerous experimental measurements probing vibrational energy and thermal transport in molecules, which indicate absence of thermalization and often ballistic heat transport [54,55,60,102,103,104]. Of course, a molecule at a junction, such as the alkane chain illustrated in Figure 4, is not isolated. Still, we may ask if the molecule were isolated whether it would thermalize under its own dynamics, or if, for energies corresponding to thermal energies in experiments, thermalization does not occur? If a molecule exhibits MBL when isolated, it thermalizes only very slowly when its end-groups are in contact with its environment, and thus does not thermalize on a time that is relevant to thermal transport in a junction on the nm length scale [68]. We illustrate this here for alkane chains.

To date, thermal conductance due to molecular vibrations in a single-molecule junction has not been measured. Instead, thermal conductance of a variety of monolayer junctions has been measured, where it is often assumed that thermal transport through individual molecules occurs in parallel [105]. Bonding between molecule and substrate has been found to control thermal transport at the interface [54,63], but in studies that have controlled for bonding while varying the length of the molecule [105] conductance does not appear to change in many cases, e.g., for alkane chain monolayers, indicating that thermal transport through the molecule occurs essentially ballistically. In the absence of thermalization a quantum mechanical approach based on the Landauer method, which neglects effects of inelastic scattering and thermalization [55,106,107], may be appropriate for modeling thermal conduction. The Landauer formalism, introduced to quantify electrical conductance in mesoscopic systems [108,109], can be applied to thermal transport through molecules between two leads at different temperature. We find that this approach yields results for the thermal conductance, *h_Bd_*, where *Bd* refers to thermal conductance across a boundary, through alkane chains and fluorinated alkane chains bridging gold and sapphire that are in good agreement with experiment [60], as shown in Figure 4.

If thermalization within the molecule were rapid, the variation of the thermal conductance with change in temperature would be quite different from the variation seen in Figure 4. Phonons from gold enter the molecular layer up to a frequency corresponding to the Debye temperature of gold, about 170 K. The molecule has many higher frequency modes, but if thermalization in the molecule occurs only very slowly then only modes corresponding to about 170 K transport energy to sapphire. On the other hand, if thermalization within the junction occurs sufficiently rapidly, up-conversion of vibrational energy in the molecule to vibrational modes of higher frequency occurs, enhancing thermal conduction, more so as temperature increases. This is illustrated in Figure 4 by the dotted red curve for perfluoroalkanes between gold and sapphire, which is calculated using a model where thermalization is assumed to occur rapidly. We see a striking contrast to the results using the Landauer model, where thermalization is neglected, which fit the experimental data [60] quite well.

**Figure 5:**
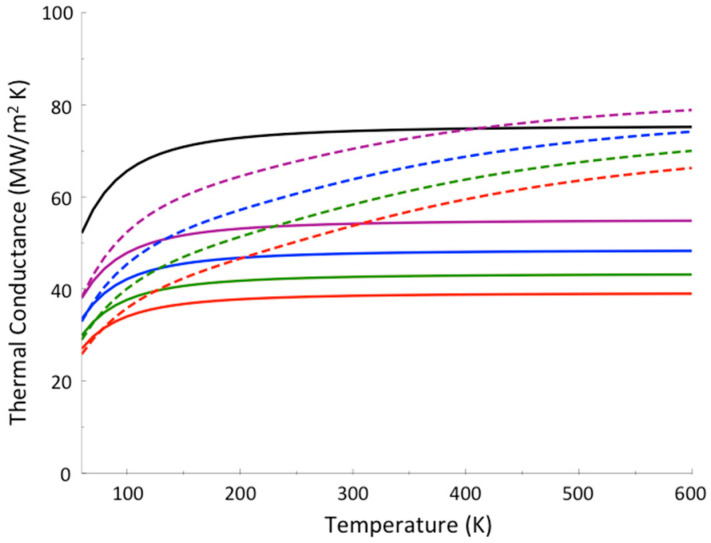
Thermal boundary conductance between Au and sapphire (black), and the thermal boundary conductance with a layer of PEG_4_ (violet), PEG_6_ (blue), PEG_8_ (green), PEG_10_ (red) between Au and sapphire, where calculations were carried out using both the Landauer model (solid curves) and a model where thermalization within the molecular junction is assumed rapid (dashed curves). Reprinted with permission from Pandey, H.D., Leitner, D.M., “Influence of thermalization on thermal conduction through molecular junctions: Computational study of PEG oligomers,” *J. Chem. Phys.* **147**, 084701, Copyright (2017), American Institute of Physics.

To further illustrate the contrast between thermal conductance when thermalization occurs and when it does not occur in the junction, we plot in Figure 5 the results of a calculation for polyethylene glycol (PEG) oligomer junctions, again between gold and sapphire. The PEG oligomers vary in length from 4 units (PEG_4_), about 1.6 nm in length, to 10 units, about 4 nm in length. Differences in predictions where thermalization does and does not occur are again striking. For these systems, separate calculations for PEG oligomers indicate that thermalization is expected to be quite rapid, and we expect thermal conductance to rise more rapidly with temperature than the Landauer model predicts [66]. Which of these trends actually occurs can be settled by the kind of experiments [60] that were carried out on alkane and perfluoroalkane chains, which produced the results plotted in Figure 4.

Thermalization in the junction effectively opens additional channels for heat transfer from gold to sapphire, and can be thought of as an analog to the RRKM theory limit for chemical reactions, where the molecule can reach the transition region from any state on the energy shell. In contrast, when thermalization in the junction is very slow, there are vibrational states in the junction that cannot be reached, which could otherwise transfer energy between the leads. For that reason, thermalization tends to enhance the rate of energy transfer across the junction and the thermal conductance.

For sufficiently slow thermalization, a calculation that neglects thermalization altogether can predict thermal conductance of a molecular junction quite well. In this respect, the question of whether or not the ETH is valid for the molecule that forms the junction is not that important. Nevertheless, quantum mechanical effects that give rise to localization in the VSS of isolated molecules can substantially reduce the rate of thermalization when the molecules form a junction. For example, an isolated alkane chain with 15 carbon atoms and energy corresponding to about 200 K is predicted to exhibit localization in the VSS. Even accounting for coupling to the leads, the rate of thermalization, below 0.1 ps^−1^, is much too slow to influence thermal conduction through the ≈ 2 nm junction [68]. Longer alkanes thermalize in ≈1 ps, fast enough that thermalization in the junction may contribute to thermal conduction.

## 5. Conclusions

We reviewed a theory for ergodicity and localization in a quantum mechanical coupled non-linear oscillator system, which models the vibrational degrees of freedom of a molecule. If a system of *N* uncoupled non-linear oscillators is perturbed by anharmonic interactions, there is a many-body localization (MBL) transition in the vibrational state space (VSS) of the molecule. This transition can occur at rather high energy in a sizable molecule. If the Hamiltonian includes only cubic anharmonic interactions, the density of states coupled by cubic anharmonic terms scales as *N*^3^, in marked contrast to the total density of states, which scales as exp(*aN*), where *a* is a constant. The locality of coupling is due to the selection rules that arise from the anharmonic interactions. Selection rules have been incorporated into random matrix ensembles as needed for a long time [42,110,111,112]. In this case, a random matrix ensemble that captures the locality of coupling in the VSS due to the selection rules imposed by the order of anharmonicity, which we refer to as local random matrix theory (LRMT), gives rise to the emergence of a MBL transition in the VSS (Fock space) of this system. LRMT has been generalized to include anharmonicity of arbitrary order and applied to locate the MBL transition for many molecules [9]. We have provided an illustrative calculation here that reveals that for the ETH to be valid, even a sizable molecule, with tens of atoms, may require energy that exceeds the energy to surmount a barrier to reaction. 

Because the ETH is often not valid for even large molecules at energies corresponding to a barrier to reaction or higher, simple theories to predict chemical reaction rates, such as microcanonical transition state theory (TST), where equilibrium statistical mechanics is adopted to predict the reaction rate, are found to break down. We have illustrated that here with the example of *trans*-stilbene isomerization, which has been very well studied experimentally. Numerous other reactions where the standard TST predictions break down have been reviewed recently [9]. The recognition that chemical reactions are more complex than can be captured by traditional theories based on equilibrium statistical mechanics, and the development of new approaches to this problem, accounting for complex dynamics in the VSS of a molecule, continue to be an active research topic [44,45,113,114,115,116,117,118,119,120,121,122,123,124,125,126,127,128,129,130]. We have also reviewed here some recent work examining the role of thermalization in thermal conduction in molecular junctions.

Computational studies of coupled many-oscillator systems [131], reviewed elsewhere [9,132], support the picture presented here, and we expect future computational work will further probe the localization transition in the VSS of molecules and the validity of the ETH. We also anticipate that the MBL transition in molecules will be further tested by experimental and computational studies of the vibrational states of cold molecules and their chemistry in cold environments [133,134,135,136,137,138,139,140,141,142,143,144,145,146,147,148,149,150,151,152,153,154,155].
